# Supplementary material for: Lysergic acid diethylamide induces increased signalling entropy in rats’ prefrontal cortex
Source: J Neurochem. 2021 Nov 14;162(1):9–23. doi: 10.1111/jnc.15534 (PMC9298798; doi:10.1111/jnc.15534)
Supplement: Supplementary file 1 — Supplementary Material [file JNC-162-9-s004.pdf]

Supplementary material

"LSD induces increased signalling entropy in rats' prefrontal cortex"

Aurora Savino, Charles D. Nichols

Supplementary Figure 1. Changes in signalling entropy (A, B) and number of splicing junctions used (C) do not depend on sequencing depth. Results shown have been obtained through subsampling of original sequencing data to the same depth of 10 million reads. N = 10, number of rats per treatment group.

Supplementary Figure 2. Modules regulated upon LSD treatment in the DOI treatment dataset. Significance is obtained with the Wilcoxon rank sum test. * = 0.05, ** = 0.01, *** = 0.001, **** = 0.0001. N = 4, number of mice per treatment group.

Supplementary Figure 3. Tcf4 regulation in the DOI (A, n = 4 mice per treatment group) and acute LSD treatment (B, n = 3 number of samples per group, each comprising two pooled rats' PFCs) datasets. Significance is obtained with the Wilcoxon rank sum test. * = 0.05, ** = 0.01, *** = 0.001, **** = 0.0001.

Supplementary Figure 4. Modules regulated in the acute LSD treatment dataset. N = 3 number of samples per group, each comprising two pooled rats' PFCs. Significance is obtained with the Wilcoxon rank sum test. * = 0.05, ** = 0.01, *** = 0.001, **** = 0.0001

Supplementary Table 1. Differentially expressed genes upon treatment with LSD

Supplementary Table 2. Gene Ontology categories enrichment for up-regulated genes after treatment with LSD.

Supplementary Table 3. Gene Ontology categories enrichment for down-regulated genes after treatment with LSD.

Supplementary Table 4. List of genes belonging to each co-expression module.

Supplementary Table 5. Gene Ontology Biological Processes enrichment for co-expression modules' genes.

Supplementary Table 6. Gene Ontology Molecular Function enrichment for co-expression modules' genes.

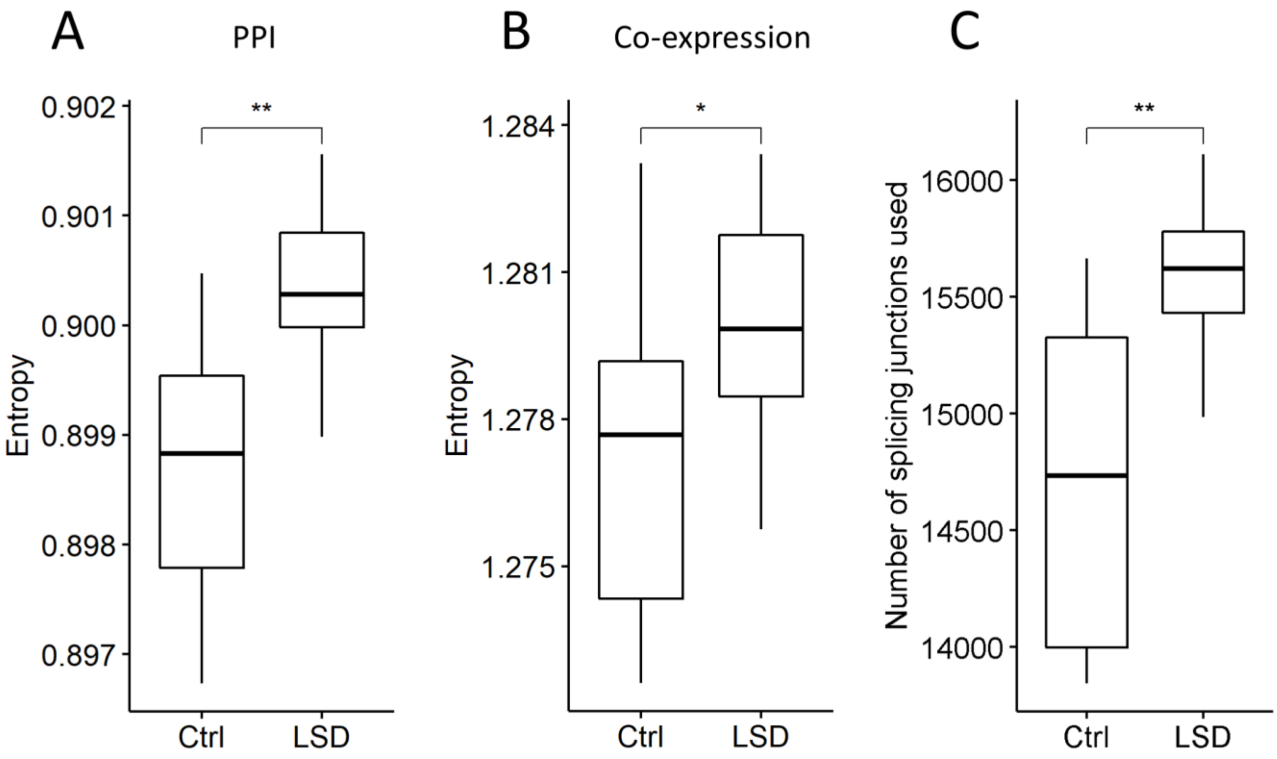

Supplementary Figure 1.

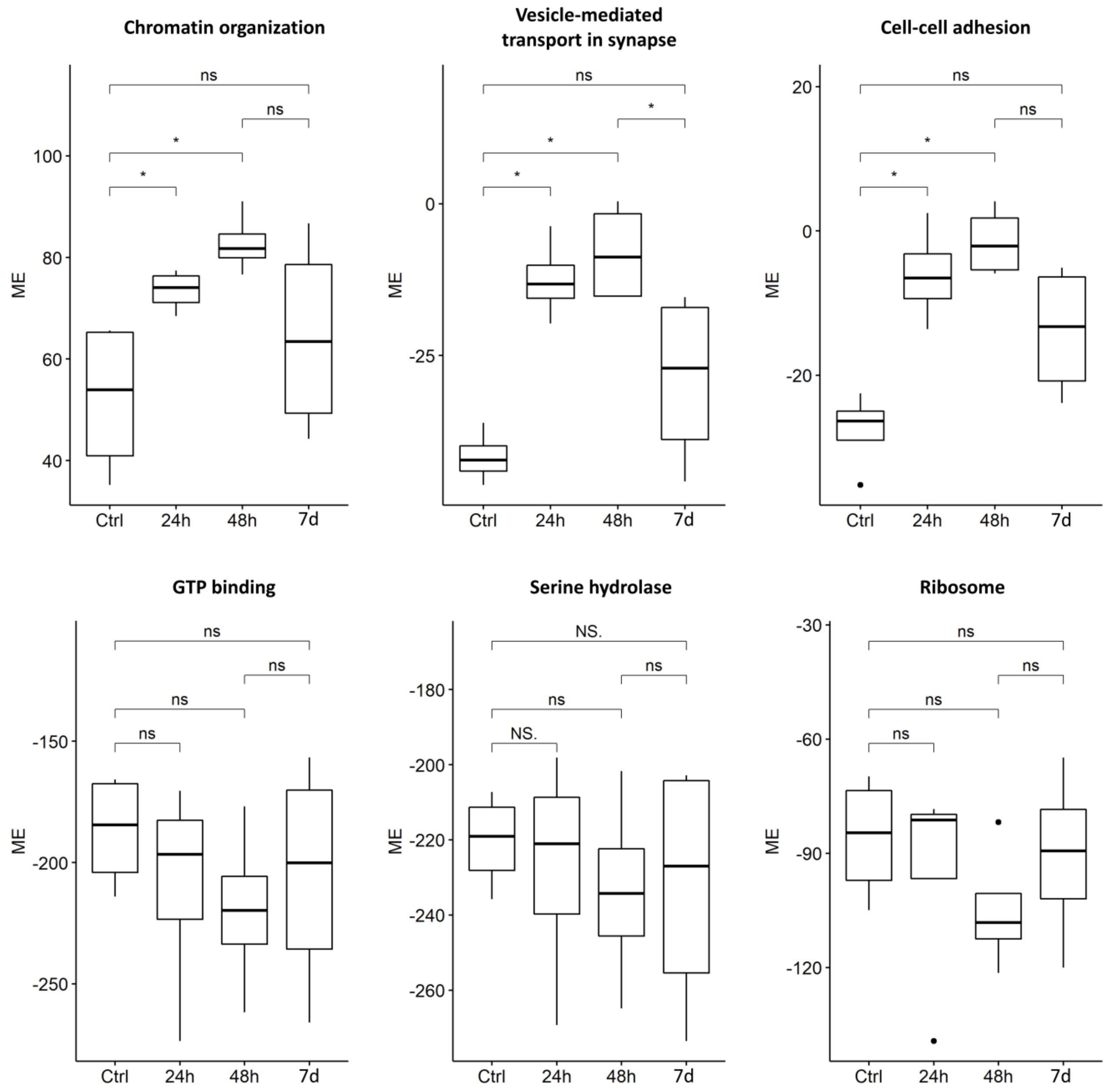

Supplementary Figure 2.

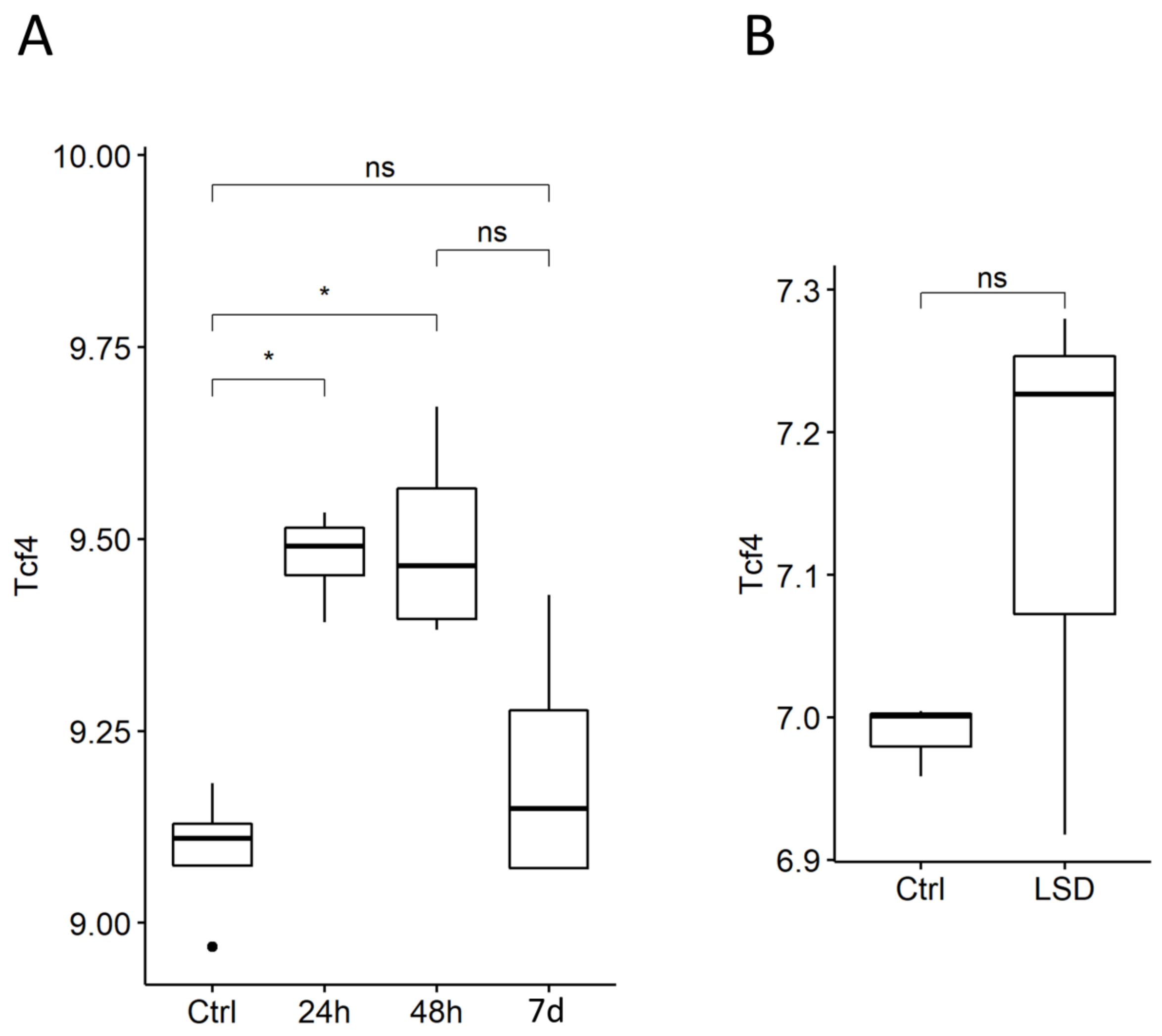

Supplementary Figure 3.

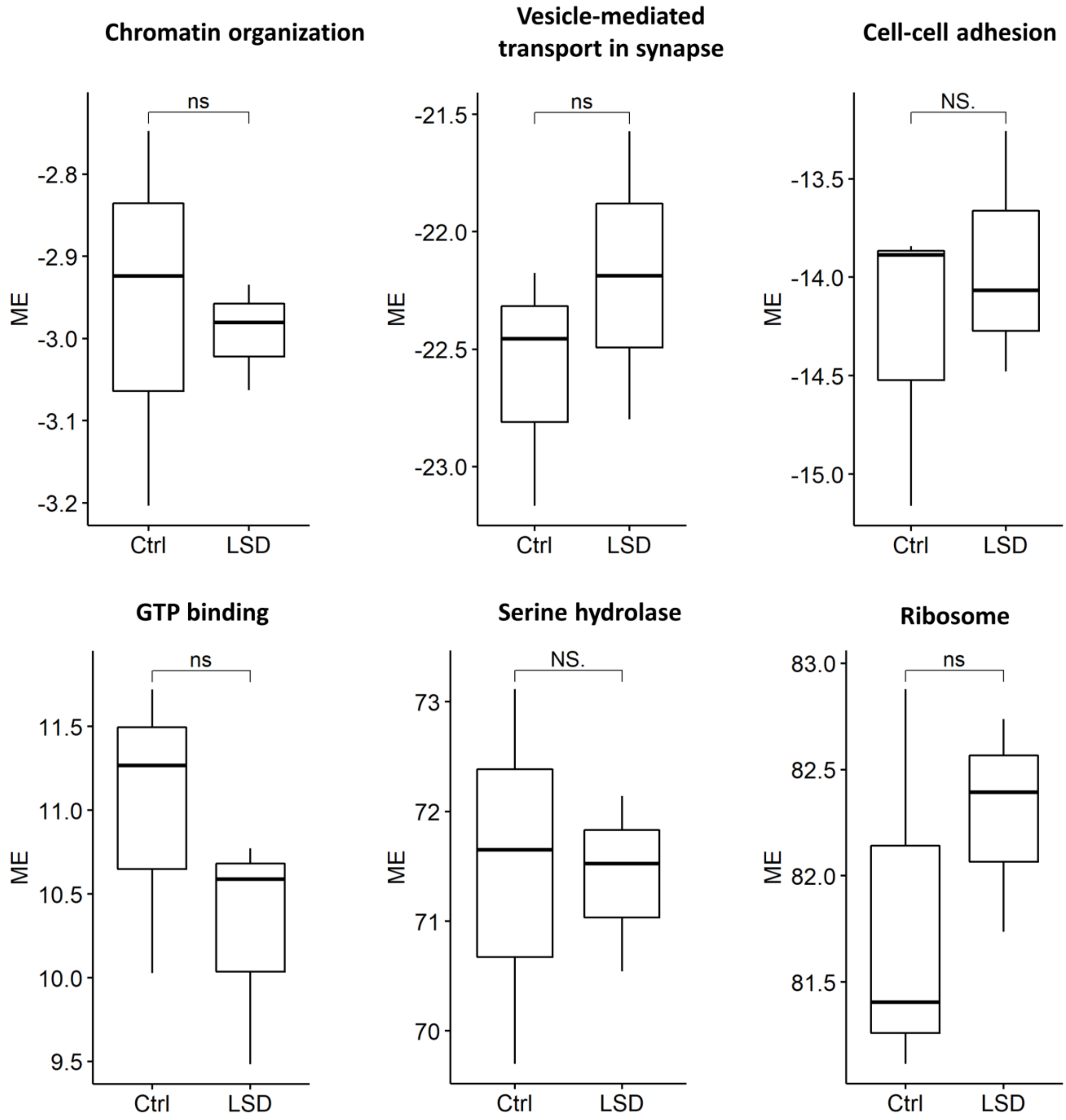

Supplementary Figure 4.
